# Brain Processing of Visual Stimuli Representing Sexual Penetration versus Core and Animal-Reminder Disgust in Women with Lifelong Vaginismus

**DOI:** 10.1371/journal.pone.0084882

**Published:** 2014-01-22

**Authors:** Charmaine Borg, Janniko R. Georgiadis, Remco J. Renken, Symen K. Spoelstra, Willibrord Weijmar Schultz, Peter J. de Jong

**Affiliations:** 1 Department of Clinical Psychology and Experimental Psychopathology, University of Groningen, Groningen, The Netherlands; 2 Department of Neuroscience, section Anatomy, University Medical Centre Groningen (UMCG), Groningen, The Netherlands; 3 Department of Neuroscience, Neuroimaging Centre (BCN-NiC), University Medical Centre Groningen (UMCG), Groningen, The Netherlands; 4 Department of Obstetrics and Gynecology, University of Groningen, University Medical Center Groningen (UMCG), Groningen, The Netherlands; University of Leicester, United Kingdom

## Abstract

It has been proposed that disgust evolved to protect humans from contamination. Through eliciting the overwhelming urge to withdraw from the disgusting stimuli, it would facilitate avoidance of contact with pathogens. The physical proximity implied in sexual intercourse provides ample opportunity for contamination and may thus set the stage for eliciting pathogen disgust. Building on this, it has been argued that the involuntary muscle contraction characteristic of vaginismus (i.e., inability to have vaginal penetration) may be elicited by the prospect of penetration by potential contaminants. To further investigate this disgust-based interpretation of vaginismus (in DSM-5 classified as a Genito-Pelvic Pain/Penetration Disorder, GPPPD) we used functional magnetic resonance imaging (fMRI) to examine if women with vaginismus (n = 21) show relatively strong convergence in their brain responses towards sexual penetration- and disgust-related pictures compared to sexually asymptomatic women (n = 21) and women suffering from vulvar pain (dyspareunia/also classified as GPPPD in the DSM-5, n = 21). At the subjective level, both clinical groups rated penetration stimuli as more disgusting than asymptomatic women. However, the brain responses to penetration stimuli did not differ between groups. In addition, there was considerable conjoint brain activity in response to penetration and disgust pictures, which yield for both animal-reminder (e.g., mutilation) and core (e.g., rotten food) disgust domains. However, this overlap in brain activation was similar for all groups. A possible explanation for the lack of vaginismus-specific brain responses lies in the alleged female ambiguity (procreation/pleasure vs. contamination/disgust) toward penetration: generally in women a (default) disgust response tendency may prevail in the absence of sexual readiness. Accordingly, a critical next step would be to examine the processing of penetration stimuli following the induction of sexual arousal.

## Introduction

After being labelled as the ‘forgotten emotion of psychiatry’ [1], disgust has increasingly been given the spot light in the context of psychopathology. Thus far, the majority of clinical disgust research focused on its role in various types of anxiety disorders, such as specific phobia [2], obsessive compulsive disorder [3], and post-traumatic stress disorder [4]. More recently, it has been suggested, that disgust might also be a factor in certain forms of sexual dysfunctions [5]–[6]. As a prominent example, it was theorized that disgust may also contribute to the inability to have penile-vaginal penetration. This inability is characteristic for primary/lifelong vaginismus, which perhaps is the most perplexing and poorly understood of all the female sexual dysfunctions [7].

Considering the functional properties of disgust and its phenomenology, it seems highly conceivable that indeed disgust might be involved in vaginismus. It has been proposed that disgust evolved as a first line of defence to protect humans from contamination by infectious agents [8]–[9]. Through eliciting the overwhelming urge to withdraw from the disgusting cue, it would facilitate the avoidance of physical contact with and/or ingestion of pathogens. The physical proximity, body apertures, and exchange of bodily fluids that are implied in sexual behaviour provide ample opportunity for the transmission of pathogens to occur [10]. Sexual behaviour thus represents an obvious threat for passing on illness or cause disease. It is therefore highly plausible that disgust may arise during sexual intercourse. Accordingly, it has been proposed that the involuntary contraction (i.e., flinching) of the pelvic floor muscles that typifies vaginismus may be elicited by the (implicit) prospect of penetration by a potential contaminant [11]. This response is possibly part of a general defence mechanism elicited in the context of a physical threat [12]–[13]. A disgust-based interpretation of the *vaginistic* response would also be consistent with recent views that framed vaginismus as a specific phobia of sexual penetration [14]–[15]. In other words, the *vaginistic* response may essentially reflect a fear of physical contact with disgusting stimuli [16], precluding sexual penetration.

Supporting the view that disgust might indeed be somehow involved in vaginismus, women inflicted with this disorder display a generally enhanced disgust *propensity* (i.e., the frequency of responding with disgust to any stimulus) [17] and heightened self-reported sexual disgust compared to women without sexual problems [18]. As more direct evidence for the alleged role of disgust in vaginismus, recent research demonstrated that specifically in women with vaginismus, pornographic film clips elicited facial disgust responses, as indexed by electro-myographical activity of the levator labii superioris muscle [19]. As an important next step to test the alleged role of disgust in vaginismus, the present study was designed to examine whether in women with vaginismus the central (brain) processing of sexual penetration stimuli would show a relatively strong convergence with the brain processing of generally disgust-eliciting stimuli.

Previous brain imaging studies employing less explicit erotica than sexual penetration have shown that also in sexually asymptomatic women (and men) there is already considerable convergence/overlap in brain responses towards pictures displaying sex and pictures depicting disgusting stimuli [20]–[22]. This led these authors to suggest that general arousal and/or attention phenomena may be an important connecting factor [21]. We recently reported a similar overlap in the central processing of visual stimuli representing disgust and very explicit sexual stimuli depicting sexual penetration in sexually asymptomatic women. The overlap in the central processing of sexual penetration and disgust stimuli was found to involve the bilateral occipitotemporal cortex, the right superior parietal lobule, bilateral dorsal midbrain, basal forebrain, posterior thalamus, and the right amygdala. Most important for the current context, we found that implicit and explicit disgust associations with sexual penetration stimuli could account for much of this convergence/overlap [23]. In other words, the stronger the disgust-eliciting potency of the penetration stimuli the more pronounced the overlap in the brain networks activated in response to sex and disgust stimuli. Thus the convergence in brain processing may not only reflect more general similarities in terms of arousal and saliency, but also more specific similarities with regard to their disgust-eliciting properties.

Clearly, the disgust-associations with sexual stimuli vary across individuals and has been shown to be especially pronounced in women with primary vaginismus or dyspareunia [24], [19]. This pattern of findings thus led to the current hypothesis that the overlap in brain areas activated in response to sexual penetration and disgust would be especially pronounced in those who have a problem with sexual penetration (e.g., vaginismus). When contrasting the brain networks involved in the processing of pictures representing sexual penetration versus disgust, it seems important to take the type of disgust elicitors into consideration. Most relevant for the present context, psychometric studies found consistent evidence for differentiating between core and animal-reminder (A-R) disgust elicitors [3]. Decaying food and faeces are prototypical examples of core disgust stimuli, whereas injury and mutilation are prototypical A-R disgust stimuli. It has been demonstrated that core and A-R disgust elicitors are associated with distinct patterns of behavioural avoidance, different psychopathologies [25]–[26], and with differences in brain processing [27]–[30]. Specifically, we earlier showed that activity in the right ventrolateral occipitotemporal cortex was inclined towards A-R, and that this area expressed functional connectivity that was selectively modulated by trait disgust [27]. Thus, it would seem important to separate A-R and core disgust, and to study their respective effect in women with vaginismus. We therefore included both classes of disgust elicitors in the design of this study.

To control for the influence of presenting negative stimuli per se, we also included a set of threatening stimuli in the design. To examine whether the effects are specific for women with primary (lifelong) vaginismus, or would reflect a more general phenomenon for women with sexual pain disorders (as defined in the fourth edition of Diagnostic and Statistical Manual of Mental Disorders; DSM-IV-TR, [31]), we not only included women with primary vaginismus but also women with dyspareunia. It needs mention that in the current version of the DSM (i.e., DSM-5, [32]), vaginismus and dyspareunia are combined into *Genito-Pelvic Pain/Penetration disorders* (GPPPD), yet as a critical difference, intercourse may still be possible in dyspareunia (though painful), whereas in vaginismus sexual penetration is by definition impossible [33].

To recapitulate, the present fMRI paradigm was designed to test whether i) women with vaginismus have stronger activity in response to visual stimuli representing sexual penetration in areas known to express sex-disgust overlap; ii) disgust- and sexual penetration-related brain networks overlap *most* in women with vaginismus; iii) the overlap of the disgust- and the sexual penetration-related brain network is restricted to one specific class of disgust elicitors or if it is evident for disgust elicitors in general.

## Materials and Methods

### Participants

Sixty-nine women participated in this study against modest reimbursement (i.e., forty Euros), seven volunteers were excluded from evaluation for a number of reasons. Basis for exclusion mainly included, excessive head motion, catastrophic feelings during scanning and poor compliance. The Medical Ethical Committee of the University Medical Center in Groningen (METC2009.068) approved the experiment, and all procedures were conducted in accordance with its standard during the entire project (2009–2013). All participants were asked to sign a written informed consent before inclusion in the study groups.

Our sample consisted of three groups: i) women diagnosed with primary (lifelong) vaginismus (n = 20, Mean_age_ = 25.3 years, SD = 4.4), ii) women without sexual complaints (n = 21 Mean_age_ = 23.0, SD = 1.9) and, iii) women diagnosed with dyspareunia (n = 21, Mean_age_ = 23.1, SD = 3.9). Most women with vaginismus and dyspareunia were yet untreated gynaecological outpatients of the University Medical Centre Groningen. These women were informed about our study, and when interested to participate, they could contact the research team to be screened over the phone for eligibility. A minority of the clinical groups (n = 5) had not yet attended the gynaecological clinic, but responded to the general advertisements we used for recruitment. These women self-reported to suffer from vaginal pain. All participants in the clinical groups were examined by an experienced gynaecologist (annex sexologist) to ensure/confirm that the women met the diagnostic criteria before they were screened over the phone for the present study.

The general recruitment procedure involved placing leaflets in public places (e.g., libraries and supermarkets) and advertisements in local media (e.g., in women's magazines, websites, and newspapers). Women with a history of neurological or psychiatric problems, severe head trauma, drug abuse, and/or prescribed psychotropic medications were excluded. Women could only participate if they were involved in a heterosexual relationship (for a minimum of 6-months). Those women who self-reported as not suffering from vaginismus or dyspareunia (i.e., no vaginal pain, or complaints) were not examined by the gynaecologist. These ‘healthy controls/sexually asymptomatic women’ were, however, screened over the phone and only those who had experienced sexual intercourse, and were indeed free of sexual complaints could participate. Groups were recruited (and scanned) in an alternating/interleaved fashion.

The participants were scanned in the first half of their menstrual cycle and never during menstruation. With the exception of three participants who were predominantly left handed, all participants were exclusively right handed according to the Edinburgh Handedness Inventory [34]. There was no significant variation in terms of age and educational level between the three groups (p>0.08). All women were Caucasian and fluent in the Dutch language. Participants reported moderate alcohol consumption at most, specifically no one of the participants exceeded the 12 units of alcohol per week.

### Diagnostic boundaries

For our sample, the diagnosis of vaginismus was based on the criteria formulated by the international consensus committee [35], namely, ‘*persistent or recurrent difficulties to allow vaginal entry, where structural or physical abnormalities were ruled out during the physical examination*’. The clinical interview included questions about vaginal entry, e.g. the extent to which a woman tried and succeeded to insert a finger, penis or any other object (e.g., tampon) in her vagina. Followed by a thorough (medical) history, the diagnostic procedure included a physical examination.

To have a sense of control over the examination, the women were informed that they had full autonomy to terminate the gynaeco-sexological examination at any time. The gynaecologist started by guiding the women through an anatomical description of their genital area, and once assisted to relax, they were asked to press against the gynaecologist finger, placed on the hymen. At this stage the exam was usually terminated due to over-activity of the pelvic floor muscles and/or involuntary guarding behaviour. Inclusion in the vaginismus group was only possible when during the examination attempts to insert a finger into the vagina elicited an involuntary guarding reaction, and a report of state fear at the attempt (or even the thought) of vaginal penetration. For the inclusion this guarding-avoidance behaviour had to be present also outside the clinic on attempts of penetration, together with a history of no previous vaginal penetration. To have a highly homogenous cohort, no women included in the vaginismus group had a diagnosis or co-morbidity of provoked vestibulodynia (PVD). For the (acquired/lifelong) dyspareunia group the selection criteria were persistent or recurrent pain in at least 50% of attempted or complete vaginal penetrations, with a duration of six months or more. Both deep (*pain felt deep inside the pelvis during penetration*) and superficial (*pain felt at the introitus*) dyspareunia were included. In this latter group, PVD characterized by ‘*severe, burning/sharp chronic pain that occurs in response to pressure localized to the vulvar vestibule*’ [36], was a common underlying problem.

### Stimuli

The stimuli used for scanning consisted of 36 colored photographs representing six emotional categories: ‘Neutral objects’ (NEU) (basic and not emotionally loaded objects e.g., a mug); ‘Fear’ (imminent threat, e.g. a man attacking a woman with a sharp knife against her neck) (FEAR); Core disgust (CORE) (primal disgust e.g. a person vomiting); Animal-Reminder (A-R) disgust (e.g. mutilation); ‘Sexual Penetration’ (PEN) (e.g. coital interaction, with explicit focus on penile-vaginal penetration), and ‘Interacting bodies’ (BOD) (i.e. minimally clothed men and women interacting without sexual connotations, e.g. yoga exercises). Except for NEU, all categories had a strong emphasis on bodies or bodily features. Stimuli for NEU category were chosen from the International Affective Picture System (IAPS) [37], whereas non-IAPS stimuli (all other categories) were collected by the research team in a pre-structured process. Based on characteristics agreed on a priori the research team selected 50 photographs. These characteristics included: no focus on faces; Caucasian heterosexual couples; easily recognizable features; very limited context. Selected pictures were then sent for further validation conducted with 40 women via an online survey (www.esurveyspro.com). This was done over and above the researchers' team selection to make sure that each stimulus from the relevant category elicited significantly more the intended emotion than the other categories. For example, the disgust stimuli had to elicit significantly more disgust than the other categories. The research team matched the scenes for physical features such as complexity, brightness, contrasts, and color. Apart from content-based validation, the stimuli were also validated with respect to color. No significant differences were found on the RBG color distribution (p>.2).

Stimuli were presented in a block design, with each block consisting of 10 pictures representing the same category. Each photograph was presented for 1.4 s, with a 1 s interval between consecutive stimuli. Six blocks (split by 16 s inter-block intervals), corresponding to the six stimulus categories, were run in a pseudo-randomized sequence. Six of these functional runs were acquired for each participant, separated by 30 s intervals, adding up to a total duration of the fMRI experiment of 1458 s. A psychtoolbox (http://psyctoolbox.org) application was developed for presentation of the experimental design.

Preceding the experiment a ‘training’ task was performed inside the scanner. Participants were instructed to look at the pictures presented without suppressing their responses. Given the passive nature of the design, participants were asked to respond (i.e., press a button) to an asterix ‘*’ that was over-imposed on a (fixed) randomly-selected number of photographs. These responses were recorded and used to exclude subjects, as an indication of not complying with the instructions of the study (n = 2), but were not used in the analysis. Post scanning, participants were presented with Visual Analogue Scales (VAS) to also rate the subjective appraisal of all the stimuli presented during the fMRI scanning. The VAS-ratings were implemented on a computer screen and had a scale of 0 to 100, with high scores indicating higher affect (pleasure/disgust/fear).

### Image acquisition

Images were acquired on a Philips Intera 3T MR-scanner. A sense 8-channel head coil was used for radio frequency reception. A series of echo planar imaging (EPI) volumes were acquired to measure the blood oxygen level dependent (BOLD) effect, which entailed a T2*-weighted gradient echo sequence with a repetition time (TR) of 2000 ms, and an echo time of 30 ms. Flip angle was 70 degrees using whole-brain acquisition (matrix size 64×64 voxels) and interleaved slice acquisition order, with an inter-slice gap of 0 mm and plane thickness of 3 mm. EPIs were acquired at 3×3 mm in-plane resolution. The (axial) images (volumes) were acquired parallel to the anterior-posterior commissure plane. In total 740 volumes were obtained per participant. A T1-weighted anatomical MRI (TR = 9 ms, TE = 3.5 ms, matrix size 256×256) and two diffusion tensor imaging (DTI) volumes of 55 slices each of 620 ms duration (with scan resolution of 96×96, flip angle 70 degrees) were acquired after the EPI runs. The DTI measurements were not included for this manuscript.

### Image pre-processing

For image pre-processing and analysis we used Statistical Parametric Mapping software (SPM8; University College London, UK; url: http://www.fil.ion.ucl.ac.uk). For each participant, all EPI volumes were realigned to the first volume acquired, and a mean EPI image was created. The realignment parameters were inspected and if movements exceeded 2 mm in any direction the participant was excluded from further analysis. The anatomical (T1) scan was co-registered to the mean-EPI image, and subsequently all EPI images and the T1 image were spatially normalized to MNI (Montreal Neurological Institute) standard stereotactic space [38]. Data were re-sampled to 2×2×2 mm (8 mm^3^) isotropic voxels. All volumes were smoothed with an isotropic Gaussian kernel of 8 mm full-width at half-maximum.

### Statistical analysis, 1^st^-level

After pre-processing, analyses were performed using the general linear model (GLM) and random effects models for second-level analysis [38]. First, we computed a GLM for each participant, which included regressors for the six conditions (*including conditions of no interest*) and also one for the inter-run instructions, convolved with a canonical hemodynamic response function. Rotational and translational head movements were added as nuisance variables (6 covariates). For each voxels a high-pass filter (cut-off 128 s) was applied to remove low-frequency noise from the fMRI time series. The standard procedure of excluding low-intensity voxels (implicit masking) was used. The following contrasts were computed: CORE>NEU, A-R>NEU, PEN>NEU, FEAR>NEU, BOD>NEU.

### Statistical analysis, 2^nd^ –level

To assess hemodynamic changes at the group level, these weighted contrasts (contrast images) were entered into three separate second-level flexible factorial models for the main effect of group (A), the main effect of condition (B), and for the interaction between group and condition (C). For models A and B we specified one factor (“Group” and “Condition”, respectively) with three and five levels corresponding to the three experimental groups and five stimulus categories, respectively. For model C - we entered these two factors, together with an additional factor, “Subject”. We specified one main effect (Subject) and one interaction (Group×Condition). The explicit factor Subject accounted for inter-individual differences in global BOLD activity. The factor settings were independence “yes”; variance “unequal” for factor “Group”, independence “no”; variance “equal” for “Condition”, and independence “yes”; variance “equal” for “Subjects”.

All main and interaction effects were tested at p<0.05 FWE corrected for multiple comparisons, or at p<0.001 uncorrected in case of an a priori hypothesis about involvement of specific brain areas. Our primary interest concerned the way women processed the images of penile-vaginal penetration, in this manuscript referred to as PEN-stimuli. First, we computed the difference between PEN>NEU and BOD>NEU activation maps. This was done for all women, as well as per group. Between-group differences in the processing of PEN were assessed using the same contrasts in the interaction model.

Next, we computed by means of conjunction analysis (*global null hypothesis*) the shared activity between PEN-related and disgust- and fear-related brain activity in each of the subject groups, using the following contrasts: [(PEN>BOD) ∧ (CORE>BOD)]; [(PEN>BOD) ∧ (AR>BOD)]; [(PEN>BOD) ∧ (FEAR>BOD)]. To investigate the consistency of the shared activity over groups, the ‘conjunction of conjunctions’ was calculated, i.e. [(PEN∧COREvaginismus) ∧ (PEN∧COREdyspareunia) ∧ (PEN>COREsexually asymptomatic women)]. All conjunctions were tested at p<0.05 FWE corrected for multiple comparisons.

## Results

### Subjective Evaluation of the Stimuli

**Table 1** illustrates the subjective evaluation of each stimulus-type on the dimensions of disgust, fear, and pleasure for the groups.

**Table 1:** Subjective evaluation of the stimuli as a function of group.

	Vaginismus			Dyspareunia			Healthy Controls		
	N = 20			N = 21			N = 21		
	Dimension of emotions elicited								
	Disgust	Fear	Pleasure	Disgust	Fear	Pleasure	Disgust	Fear	Pleasure
Stimuli	*m(sd)*	*m(sd)*	*m(sd)*	*m(sd)*	*m(sd)*	*m(sd)*	*m(sd)*	*m(sd)*	*m(sd)*
*CORE*	79(15)	25(28)	3(4)	82(15)	27(29)	3(4)	79(14)	27(19)	5(7)
*A-R*	82(19)	38(36)	5(6)	87(14)	47(34)	4(5)	87(17)	56(29)	4(7)
*FEAR*	50(28)	68(24)	8(8)	47(27)	75(*24*)	7(9)	40(27)	71(20)	5(6)
PEN	42(33)	28(26)	22(18)	44(31)	21(23)	23(25)	26(26)	10(12)	37(25)
*BOD*	1(2)	3(5)	57(23)	3(4)	3(6)	60(22)	1(1)	2(2)	57(19)
*NEU*	.3(.3)	.4(.4)	56(28)	.3(.4)	.5(.5)	46(25)	.6(.6)	.4(.5)	32(27)

Y-Axis, the stimuli presented on a visual analogue scale (VAS) off-magnet, X-Axis, emotions elicited on 3 dimensions (i.e., disgust, fear, pleasure) for the three groups (i.e., vaginismus, dyspareunia and healthy controls). DIS, core disgust elicitors, A-R, animal-reminder disgust elicitors; FEA, fear related stimuli; PEN, explicit sexual penetration stimuli; BOD, neutral bodies; NEU, neutral objects. The VAS had a scale of 0 to 100, with high score indicating higher affect (pleasure/disgust/fear).

For the validation of the stimulus material and relevant group differences, we ran two manipulation tests: for manipulation test (i) we investigated whether A-R and CORE disgust differ in how they were rated, when contrasted with FEAR and if there were differences between groups. A 3 *Picture* (A-R, FEAR, CORE)×3 *Emotion* (pleasure, disgust, fear)×3 *Group* (Vaginismus, Dyspareunia, Sexually asymptomatic/Controls) mixed between-within subject ANOVA was conducted. In line with expectations *pictures* elicited a differential pattern of emotional ratings, as was evidenced by the significant interaction of Picture*Emotion, Wilk's λ = .11, F(4, 56) = 106, p<.001, η = .84. This pattern did not vary across groups as evidenced by the non-significant 3-way interaction of Picture*Emotion*Group, Wilk's λ = .82, F(8, 112) = 1.50, p>.05, η = .09. We further decomposed the 2-way interaction Picture*Emotion, by conducting a (4) series of t-tests: Attesting to the validity of the stimulus materials, participants rated both A-R and CORE higher in disgust than FEAR, t(61) = 12.6, p<.001, and t(61) = 10.3, p<.001, respectively. When directly comparing both types of disgust categories it appeared that A-R elicited slightly higher disgust and also higher fear ratings than CORE, t(61) = 3.5, p = .001 and t(61) = 9.1, p<.001, respectively.

To verify if PEN stimuli were rated differently across groups and to examine how this related to both neutral contrasts (BOD and NEU), we conducted our 2^nd^ manipulation test (ii) a 3 *Picture* (PEN, BOD, NEU)×3 *Emotion* (pleasure, disgust, fear)×3 *Group* (Vaginismus, Dyspareunia, Controls) mixed between-within subject ANOVA. A significant interaction was noted for Picture*Emotion Wilk's λ = .27, F(4, 56) = 38.7, p<.001, η = .73 indicating that the pattern of ratings generally varied across stimuli with relatively high pleasure ratings for BOD and NEU together with relatively high disgust and fear ratings for PEN. This pattern varied across groups as evidenced by the significant 3-way (Emotion*Picture*Group) interaction (see also Table 1.) Wilk's λ = .70, F(8, 112) = 2.8, p<0.008, η = .17. To further investigate the 3-way interaction, we investigated the 2-way interaction Emotion*Group for each of the 3 stimuli categories (NEU, BOD, PEN).

For PEN stimuli a significant 2-way interaction was observed (Wilk's λ = .84, F(4, 116) = 2.7, p<.03, η = .09), indicating that the pattern of ratings differed across groups with both clinical groups showing trends of higher ratings on the dimension of disgust and threat as well as less pleasure, when compared to controls (see Table 1).

To further confirm that this interaction indeed is driven by the difference between the clinical versus controls, we subjected PEN to 2 *Group* (clinical vs. control)×3 *Emotion* (disgust, fear, pleasure) ANOVA, which in line with expectations reached significance in the predicted direction (Wilk's λ = .86, F(2, 59) = 4.6, p<.01, η = .14). However, there was considerable overlap with regard to the range of ratings across groups, and also within the clinical groups there were participants who assigned only low disgust to PEN stimuli.

For BOD stimuli, the two-way interaction did not reach significance indicating that the pattern was overall similar for all groups (Wilk's λ = .96, F(4, 116) = 0.54, p>0.05, η = .02). For NEU the Emotion*Group did reach significance (Wilk's λ = .75, F(4, 116) = 4.4, p<0.01, η = .13); to decompose the interaction further we conducted a 3 one-way ANOVAs separately for each emotion-dimension. Groups did not differ on the emotion of disgust and fear (p>0.16), yet showed a differential pattern regarding their pleasure ratings (F(2, 59) = 4.0, p<0.02). Post hoc comparisons showed that only the contrast between vaginismus and controls reached significance, indicating that the vaginismus group rated the NEU stimuli as more pleasurable than the controls (p<.001).

### Visual processing of penile-vaginal intercourse main effects

**Figure 1:**
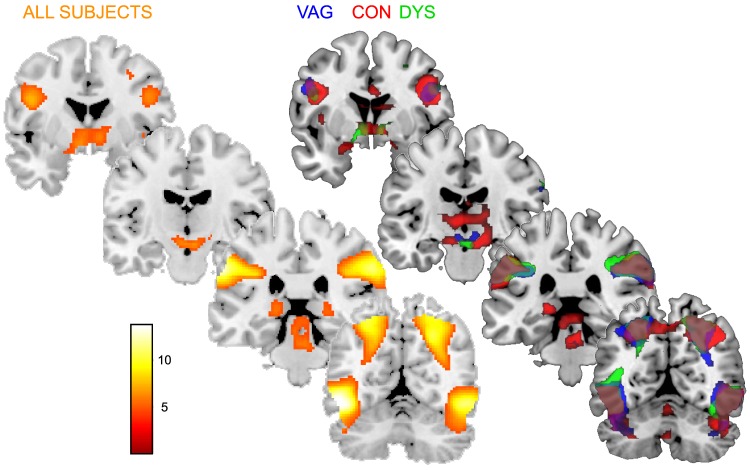
Central processing of penile-vaginal penetration images (PEN). Central processing of penile-vaginal penetration images (PEN) in all subjects (left panel), and within the three groups (right). Brain maps are thresholded at p<0.05, FWE corrected for multiple comparisons.

The PEN>BOD contrast was assumed to best capture brain activity related to the visual processing of couples engaged in sexual intercourse/penetration. Over subjects and groups significant (p<.05, FWE corrected) activity was found in widespread, bilateral occipitotemporal and occipitoparietal areas, the latter reaching up to include the superior parietal lobule (Figure 1). More rostral in the brain, activity was found in bilateral precentral gyrus corresponding to ventral premotor cortex. Substantial subcortical activity was found, centered posteriorly on the pulvinar of the thalamus and the dorsal midbrain, and anteriorly on the hypothalamus and basal forebrain (Table 2). Within the separate subject groups the overall picture was similar, although for, the sexually asymptomatic group there was more convincing activation of the subcortical areas and premotor cortex than for the clinical groups (Table 2).

**Table 2:** Central processing of penile-vaginal penetration images (PEN).

PEN>BODIES		ALL GROUPS					ASYMPTOMATIC	DYSPAREUNIA					VAGINISMUS						
		*k*	x	y	z	*T*	*k*	x	y	z	*T*	*k*	x	y	z	*T*	*k*	x	y	z	*T*
***occipitotemporal***																					
inf temp gyr	L	7431	−50	−62	−10	13,66	874	−52	−60	−10	8,18	661	−46	−64	−10	8,26	1871	−44	−64	−12	7,79
inf temp gyr	R	lm	50	−56	−12	11,74	3604	48	−58	−12	6,88	483	42	−64	−14	6,88	660	50	−56	−12	7,65
inf occ gyr	L											66	−34	−88	−4	6,59	lm	−34	−86	−6	6,74
inf occ gyr	R						lm	38	−86	−6	6,87	lm	40	−82	12	6,69	3449	40	−78	2	7,78
precuneus	M						78	0	−52	54	5,49										
***occipitoparietal***																					
supramarginal gyr (area IPC/PF)	L	lm	−60	−30	34	12,3	lm	−60	−26	34		lm	−58	−30	36		lm	−60	−32	36	6,79
supramarginal gyr (area IPC/PF)	R	8759	58	−26	36	12,31						1710	58	−24	36	8,16					
inf par lobule	L											1342	−38	−38	38	8,17	1509	−38	−46	46	7,19
inf par lobule	R											lm	34	−38	40		lm	34	−42	46	6,89
sup occ gyr/sup par lobule	L						lm	−22	−66	48	6,94	92	−28	−68	24	5,40	lm	−28	−60	54	6,67
sup occ gyr/sup par lobule	R	lm	24	−66	44	11,35	lm	28	−78	26		lm	22	−64	46						
***frontal***																					
precentral gyr/ventral premotor	L	415	−46	2	28	7,87	72	−46	2	28	5,50										
precentral gyr/ventral premotor	R	648	50	6	26	8,58	127	50	4	24	5,30	23	54	6	28	4,99	103	50	6	26	5,29
precentral gyr/dorsal premotor	L	88	−24	−6	46	5,49	21	−28	−8	46	4,96										
precentral gyr/dorsal premotor	R	105	32	−4	50	5,89															
***(para)limbic***																					
insula	R	21	42	−4	−4	5,26															
***subcortical***																					
hypothalamus/basal forebrain/vp	L	lm	−6	0	−8		6	−4	−4	2	4,63	10	−8	2	−6	5,04					
post thalamus	L	148	−18	−30	2	5,73	55	−16	−26	6	5,14										
post thalamus	R	58	22	−30	0	5,28	15	20	−24	4	4,99										
ventral thal - midbrain	R	lm	2	−16	−12	6,31															
midbrain	L	1262	−4	−24	−14	6,46	19	−8	−22	−12	4,92										
midbrain	R						8	10	−20	−14	4,86										
cerebellar hemisphere	L																				
cerebellar hemisphere	R						12	44	−64	−28	4,72										

Central processing of penile-vaginal penetration images (PEN) in all subjects, and within the three groups separately. PEN activation maps were compared with activation maps related to processing of images of a barely dressed man and woman interacting neutrally (BOD). k, number of voxels; lm, local maximum. All clusters are p<0.05, FWE corrected for multiple comparisons.

### Group differences in processing of images of penile-vaginal intercourse

No significant group differences (p<0.05, FWE corrected) were found for the (PEN vs. BOD) contrast, nor for any of the other contrasts (CORE vs. BOD, A-R vs. BOD, FEAR vs. BOD). Previously, we have found that activity in the posterior thalamus, dorsal midbrain, superior parietal lobule, occipitotemporal cortex, basal forebrain and amygdala tracked enhanced disgust responses during PEN exposure [23], but even at a lenient statistical threshold (p<0.001 uncorrected) none of these areas showed a tendency to be more activated by PEN in women with vaginismus or dyspareunia than in asymptomatic women.

### Shared brain activity from viewing penetration and aversive images

**Figure 2:**
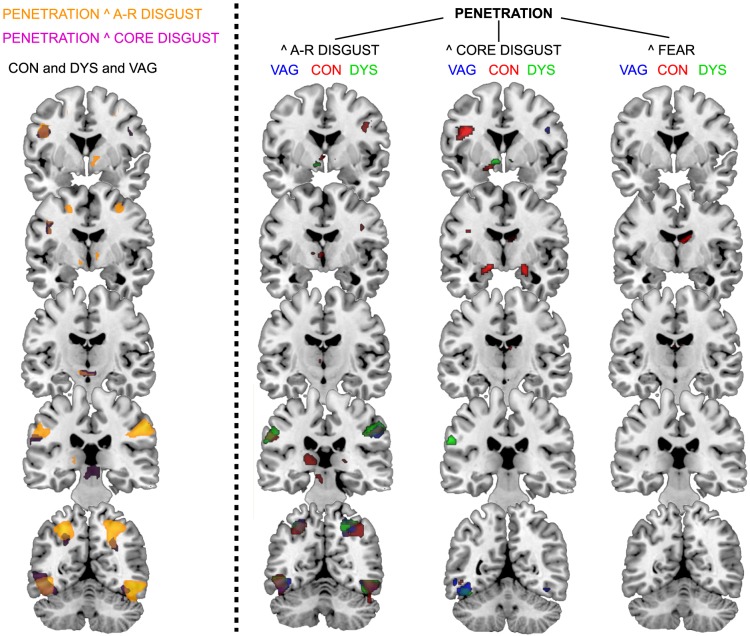
Overlap between PEN and disgust brain activation maps. Overlap between PEN and disgust brain activation maps, resulting from an analysis on the conjugated activity (PEN>BOD ∧ PEN>BOD), [(PEN>BOD ∧A-R>BOD) and (PEN>BOD ∧ FEAR>BOD). The left panel of the figure depicts the consistency of the PEN ∧A-R(orange shading) and PEN ∧ CORE (purple shading) maps across all subjects. The right panel depicts the conjugated activity within each group individually (sexually asymptomatic, red; vaginismus, blue; dyspareunia, green). Note the more extensive overlap for PEN ∧A-R with respect to the other conjunctions, especially in posterior parts of the brain. A-R, animal-reminder disgust; CORE, core disgust; PEN, penile-vaginal penetration; DYS, dyspareunia; CON, sexually asymptomatic; VAG, primary vaginismus. Brain maps are thresholded at p<0.05, FWE corrected for multiple comparisons.

No formal statistical test was performed on differences between PEN ∧ A-R and PEN ∧ CORE conjunction maps (Table 3). However, the weight of the shared activity seemed to be on the PEN ∧ A-R conjunction (appr. ten times more voxels than the PEN ∧ CORE map). For instance, only for the PEN ∧ A-R conjunction the shared activity involved occipitoparietal areas (reaching up to the superior parietal lobule). For PEN ∧ AR, sexually asymptomatic women clearly showed the most overlap (inferred from number of suprathreshold voxels), but for PEN ∧ CORE this bias was less obvious. For instance, only sexually asymptomatic women showed above threshold joint hypothalamic, posterior thalamic, and midbrain activity for the PEN ∧ A-R conjunction, and joint amygdala activity for the PEN ∧ CORE conjunction. Either way, this picture is not in line with the hypothesis that women with vaginismus would show more convergence in their brain responses to PEN and disgust. This was confirmed when the consistency of these shared effects over groups was calculated (Fig. 2, left). It became clear that consistency over groups was considerable for the PEN-disgust convergence, making it unlikely that women with vaginismus had more pronounced convergence in the brain processing of these stimuli. A dissociation was seen in the subcortex, where PEN ∧ CORE overlap was expressed in the dorsal midbrain/PAG and PEN ∧ A-R overlap more rostrally in the basal forebrain-ventral pallidum.

**Table 3:** Shared brain responses between PEN-and aversive- brain activity maps.

PEN ∧ DISGUST (A-R)		ASYMPTOMATIC					DYSPAREUNIA	VAGINISMUS
		*k*	x	y	z	*T*	*k*	x	y	z	*T*	*k*	x	y	z	*T*
***occipitotemporal***																
inf temp gyr/fus gyr	L	606	−52	−60	−10	6,12	326	−46	−66	−10	5,88	372	−44	−64	−10	5,43
inf temp gyr/fus gyr	R	573	50	−60	−12	5,42	312	44	−66	−12	4,98	349	50	−56	−12	5,36
inf occ gyr	L	lm	−32	−88	−4	3,62	47	−34	−88	−4	4,57	655	−32	−88	−6	4,71
inf occ gyr	R	2512	42	−88	−4	4,71	299	42	−80	10	4,81	1681	42	−80	4	5,50
***occipitoparietal***																
supramarginal gyr (area IPC/PF)	L	95	−60	−26	32	4,22	lm	−62	−26	30	4,62					
supramarginal gyr (area IPC/PF)	R						370	56	−26	40	5,54	148	56	−24	32	4,23
sup occ gyr/sup par lobule	L	1318	−20	−68	48	4,93	105	−28	−68	24	3,75	lm	−26	−70	28	4,11
sup occ gyr/sup par lobule	R						406	20	−66	56	4,35	588	−24	−64	54	4,36
inf par lobule	L	lm	−44	−38	40	4,39	1045	−38	−38	38	5,34	lm	−40	−36	34	4,27
inf par lobule	R	lm	48	−36	38	4,50										
***frontal***																
precentral gyr/premotor	R	38	40	−2	30	3,55						72	44	8	32	3,55
precentral gyr/premotor	L	22	−48	2	30	3,49						7	−50	4	30	3,28
***subcortical***																
hypothalamus	L	64	−4	−10	4	3,42										
hypothalamus	L	lm	−4	0	−2	3,23	20	10	0	−6	3,72					
post thalamus	L	113	−16	−26	6	3,90										
post thalamus	R	15	20	−24	4	3,57										
midbrain	L	46	−8	−22	−12	3,71										
midbrain	R	11	4	−30	−24	3,22										
midbrain	R	20	12	−20	−14	3,31										

PEN ∧ DISGUST (CORE)		ASYMPTOMATIC					DYSPAREUNIA					VAGINISMUS				
		*k*	x	y	z	*T*	*k*	x	y	z	*T*	*k*	x	y	z	*T*
***occipitotemporal***																
inf temp gyr/fus gyr	L	18	−32	−48	−18	3,24	194	−42	−46	−18	4,13	176	−46	−54	−16	3,97
inf temp gyr/fus gyr	R	19	34	−50	−16	3,23	61	38	−62	−22	3,44	59	40	−48	−16	3,50
inf occ gyr	R						3	34	−90	0	3,71	24	36	−86	−6	3,40
***occipitoparietal***																
sup occ gyr/sup par lobule	L											27	−20	−70	38	3,37
supramarginal gyr (area IPC/PF)	L						97	−62	−26	28	4,69					
***frontal***																
precentral gyr/ventral premotor	L	122	−44	4	22	3,78						7	−50	4	30	3,20
precentral gyr/ventral premotor	R											36	46	2	28	3,39
***(para)limbic***																
insula	L	lm	−36	2	26	3,61										
***subcortical***																
amygdala/vp/subthalamic	L	65	−16	−4	−12	3,59										
amygdala/vp/subthalamic	R	51	22	−6	−16	3,86										
hypothalamus	L						34	−8	2	−6	3,85					
caudate	R	49	8	−8	18	3,73										

PEN ∧ FEAR		ASYMPTOMATIC					DYSPAREUNIA					VAGINISMUS				
		*k*	x	y	z	*T*	*k*	x	y	z	*T*	*k*	x	y	z	*T*
**subcortical**																
caudate	R	81	10	−8	20	3,87										

Shared activity between PEN-related brain responses and brain responses related to aversive stimuli within the three groups. Results are shown from the conjunction analyses (PEN>BOD ∧ CORE>BOD), [(PEN>BOD ∧ A-R>BOD) and (PEN>BOD ∧ FEAR>BOD). A-R, animal-reminder disgust; CORE, core disgust; PEN, penile-vaginal penetration; k, number of voxels; lm, local maximum. All clusters are p<0.05, FWE corrected for multiple comparisons.

## Discussion

This study is the first to register brain activity in women with primary/lifelong vaginismus when exposed to disorder-specific stimuli. The major aim of this fMRI study was to test if women with vaginismus would show relatively strong convergence in their brain responses towards sexual penetration (PEN) and disgust-related pictures compared to sexually asymptomatic women and women suffering from vulvar pain (dyspareunia). The major results can be summarized as follows: (i) the brain responses to PEN did not differ between groups, (ii) there was a large overlap between the brain responses elicited by PEN and disgust pictures that seemed most elaborate for A-R disgust; however, also this conjoint PEN and disgust-related brain responses did not differ between groups.

Attesting to the validity of the stimulus material, both disgust categories (i.e., A-R, core) were rated as much more disgusting than the fearful control pictures. The finding that disgust stimuli also elicited some fear is consistent with the dominant disease-avoidance explanation of disgust [8]–[9]. The prospect of being contaminated by pathogens (either via incorporating toxic/food items, or via close physical contact with people who carry a transmittable disease), will logically also elicit fear of contamination and/or of getting inflicted by a disease [39]. The A-R disgust stimuli elicited stronger feelings of disgust and fear than stimuli representing core disgust. Although both categories of disgust elicitors are assumed to have the same functional properties of indicating pathogen transmission [6], the features of deformed body parts that make part of A-R disgust elicitors, may not only represent a stronger contamination potency than core disgust elicitors (such as food items and body waste products), but also more directly represent the (threatening) consequences of actually getting contaminated (by the pathogens responsible for the depicted condition).

The subjective ratings of the disgust stimuli were similar for all groups. Thus no evidence emerged to suggest that pictures representing core or A-R stimuli elicited relatively strong feelings of disgust in women with vaginismus. Yet, in line with previous studies [24], [40], [19], [41] women with vaginismus subjectively rated the disorder-specific PEN stimuli as more disgusting, more threatening and less pleasurable than the group of sexually asymptomatic women. A similar pattern was evident for women suffering from dyspareunia. At the brain level, however, no differences appeared between symptomatic and asymptomatic women when exposed to PEN pictures.

This lack of group differentiation was rather unexpected. First, previous research in sexually asymptomatic women has shown that the strength of PEN-induced brain activity in a number of brain areas, including the posterior thalamus and the dorsal midbrain was correlated with the strength of explicit and implicit disgust associations with PEN [23]. Second, women with vaginismus have consistently been found to show heightened disgust to PEN stimuli (relative to sexually asymptomatic women) [19]. As alluded to in the introduction, together, this would predict stronger brain activity towards PEN, as well as more overlap of PEN- and disgust- induced brain responses in women with vaginismus versus the other two groups.

Generally, women with vaginismus showed brain responses ‘typical’ for visual sexual stimulation, despite using a more specific control stimulus (i.e., barely dressed people interacting neutrally) than was previously used in other studies; namely, higher-order visual areas in the occipitotemporal cortex, areas implicated in attention like the superior parietal lobule, higher-order somatosensory areas in the inferior parietal lobule, and premotor areas [42]. Subcortical responses to PEN did not reach statistical significance in women with vaginismus, whereas they clearly did in the sexually asymptomatic women. These responses were observed in the hypothalamus, ventral pallidum, in the pulvinar of the thalamus and in the midbrain. Nevertheless, this apparent divergence was insufficient for a significant group difference in these subcortical areas, even at a lenient statistical threshold. Thus, there was no convincing difference between women with and without vaginismus with regard to their brain responses toward PEN.

The overlap between PEN- and disgust-induced brain activation was extensive with more overlap i.e., more shared voxels, observed between PEN and A-R disgust than between PEN and core disgust activation maps, across the three groups. As Figure 2 illustrates, the shared activity between disgust and PEN-induced brain responses involved the occipitotemporal cortex, the superior and inferior parietal lobule, the inferior frontal gyrus, and ventral midbrain regardless of disgust domain. Partly, however, the overlap also involved different areas in the brain; only for A-R disgust the shared activity also occurred in the hypothalamus and ventral pallidum, whereas only for core disgust conjugated activity was seen in the dorsal midbrain. Despite that both disgust elicitors are assumed to have the same function of cueing the risk of pathogen transmission, they are two different classes of disgust elicitors, and possibly these differences in overlap with PEN reflect the differences in their respective pathways of pathogens transmission.

We have previously reported on the conjugated activity between PEN and core disgust in sexually asymptomatic women of this subject cohort [23]. This shared activity was substantial, and similar to that reported by other research groups using soft (non-PEN) erotic pictures [20]–[22]. The present findings however, extend these earlier reports in three major ways: First, the overlapping brain activity with PEN is not restricted to core disgust elicitors, but seems even more elaborate for A-R disgust elicitors. Second, the current study indicates that this overlap is independent of the absence/presence of sexual problems. Third, because hardly any overlap was observed between PEN and (negative and arousing) fear-inducing pictures, the present findings show that the overlap in brain responding toward PEN and disgust stimuli cannot be attributed to more general similarities between PEN and disgust in terms of their arousing properties or general affective tone.

This overlap of brain response to PEN and disgust stimuli across groups is consistent with the idea that disgust contributes to penetration-related disorders like vaginismus. Strikingly, sexually asymptomatic women exhibit very similar - if not stronger - overlap between PEN and A-R disgust central processing. This flips the question - from ‘*is disgust involved in vaginismus?*’ to ‘*how do healthy women succeed in having pleasurable sex at all, in light of the strong disgust component apparently involved in sex?*’ [5].

It could be speculated that women hold a relatively ambivalent attitude towards cues of penetration: on the one hand, avoidance of sexual stimuli/penetration may be triggered in order to prevent contamination by pathogen transmission, whereas on the other hand approach might be triggered to support procreation and pleasure [43], [5]. The activity/responses of the hypothalamus and ventral pallidum in the PEN-A-R conjunction would then reflect their known involvement in arbitrating/deciding on approach and avoidance responding [44]–[45]. The fact that vaginal penetration without additional clitoral stimulation, though intrinsically pleasurable, does not lead to orgasm in more than half of sexually active women might help in shifting the women's appraisal towards a negative appreciation. Moreover, because this study was conducted in a laboratory (fMRI scanner) context, in the absence of sexual readiness, this negative response may have been particularly pronounced [46].

Besides, women were just passively viewing the presented stimuli and were not primed to a particular evaluative mode; this might also be relevant for the understanding of the present pattern of results (i.e., group differences did not emerge in the brain responses to these stimuli). In previous research that used reaction time tasks, participants were asked to categorise PEN stimuli on a dimension of disgust-hot, specifically sexually symptomatic women displayed strong PEN-disgust associations, whereas women without sexual complaints showed relatively strong PEN-‘hot’ associations [19]. For asymptomatic women, positive ‘hot’ associations might be more accessible due to their more extensive (probably pleasurable) experience with PEN. In other words, in asymptomatic women PEN might not only elicit the default disgust associations, but also the relatively positive-specific associations (at least when provided on a dimension such as ‘disgust-hot’), specific associations that are perhaps less accessible in sexually symptomatic women. Consistent with such an explanation, a differential pattern between (sexually) asymptomatic and symptomatic women was evident for the current subjective ratings, which were conducted in an evaluation mode [40].

The absence of an evaluation mode and/or the absence of sexual arousal might help to explain why even women without penetration-related problems showed a strong convergence in their brain response towards PEN and disgust. It has recently been put forward that sexual arousal may be a critical factor that can switch the default disgust response to a sexual appetitive/approach response [43]. Accordingly, it has been demonstrated that when sexually aroused, sexually asymptomatic women rated sex-related disgusting stimuli as less disgusting, and reduced avoidance compared to either generally aroused women or women in a neutral mood [46]. One could therefore speculate that the sexual complaints could be explained by some impairment to overrule the default sex-disgust response. Attesting to this, in contrast to asymptomatic women, women with vaginismus continued to show (facial) physiological signs of disgust while they were watching a sexually arousing erotic movie [19], [5]).

In conclusion, this paper is a first attempt to tap in the neural correlates of women with vaginismus by presenting disorder specific (penetration) stimuli. The lack of stronger brain activity convergence in response to PEN and disgust in women with vaginismus versus sexually asymptomatic women, suggests that this overlap reflects a default disgust response towards penetration stimuli across all women, perhaps more so in the absence of sexual readiness. A critical next step would be to examine the processing of PEN stimuli following sexual arousal induction.
